# Cytokine profiles among patients co-infected with *Plasmodium falciparum* malaria and soil borne helminths attending Kampala International University Teaching Hospital, in Uganda

**DOI:** 10.1186/s13223-018-0235-z

**Published:** 2018-03-19

**Authors:** Richard Bwanika, Charles D. Kato, Johnson Welishe, Daniel C. Mwandah

**Affiliations:** 1 School of Biomedical Sciences, Department of Microbiology, Kampala International University, Western Campus, Ishaka, Box 71, Bushenyi, Uganda; 2 School of Bio-security, Biotechnical & Laboratory Sciences, College of Veterinary Medicine, Animal Resources & Bio-security, Makerere University, P.O Box 7062, Kampala, Uganda

**Keywords:** *Plasmodium falciparum* malaria, Soil borne helminths, Th1 and Th2 cytokines, Co-infected

## Abstract

**Background:**

Malaria and helminths share the same geographical distribution in tropical Africa. Studies of the interaction of helminth and malaria co-infection in humans have been few and are mainly epidemiological, with little information on cellular immune responses. This study aimed to determine Cytokine profiles among patients co-infected with *Plasmodium falciparum* malaria and soil borne helminth attending Kampala International University Teaching Hospital (KIU).

**Methods:**

A case control study of 240 patients were recruited at KIU teaching hospital. Patients with *Plasmodium falciparum* malaria were 55 (22.9%) and those with soil-borne helminths were 63 (26.3%). The controls were 89 (37.1%), while those co-infected with *Plasmodium falciparum* malaria and soil-borne helminths were 33 (13.8%). Cases were defined as having a positive blood smear for *P. falciparum* malaria, those with helminths or co-infections of the two. Negative controls were those with a negative blood smear for *P. falciparum* malaria and those with no stool parasitic infections. Patients presenting with signs and symptoms of malaria or those suspected of having helminths were recruited for the study. A panel of five cytokines (IFN-γ, TNF-α, IL-6, TGF-β and IL-10) were assayed from plasma samples in patients with and without *Plasmodium falciparum* malaria, patients with and without helminth, and then those co-infected with the two diseases diagnosis was done using thick blood smears stained with 10% Giemsa and stool examination was done following the Kato Katz technique following standard procedures.

**Results:**

The prevalence of *Plasmodium falciparum* malaria by sex was 28 (11.7%) and 27 (11.3%) in male and female respectively. The overall prevalence of soil borne helminth was 26.3%, and among those harbouring helminths, 13.8% were co-infected with *Plasmodium falciparum.* Cytokine levels significantly differed across *Plasmodium falciparum* malaria, soil borne helminth infected patients and health controls for IFN-γ (P = 0.023), IL-10 (P = 0.008) and TGF-β (P = 0.0001). Cytokine levels significantly differed across *Plasmodium falciparum* malaria, soil borne helminth infected patients and patients co-infected with *Plasmodium falciparum* malaria and soil borne helminth for IL-10 (P = 0.004), IL-6 (P = 0.011) and TGF-β (P = 0.003).

**Conclusion:**

An up-regulation of IFN-γ during *Plasmodium falciparum* malaria and an up-regulation of IL-10 and TGF-β in soil borne helminth infections was demonstrated. We demonstrate that co-infections of *Plasmodium falciparum* and soil borne helminth lead to an up-regulation of IL-10 and IL-6 and a down-regulation of TGF-β.

*Trial registration* No17/10-16

## Background of this study

Malaria remains a global burden with approximately 584,000 deaths among an estimated 198 million cases annually [1]. The biggest disease burden is mainly encountered among children in sub-Saharan Africa [1, 2]. This scenario is further complicated by the overlapping distribution of parasitic diseases in the tropics [3, 4] among which malaria-helminths co-infections are common [3, 5, 6]. It is estimated that half a billion people in the developing world harbour one or multiple helminths [7]. Malaria and helminths co-infections are mainly driven by poverty, tropical environment, water bodies and poor control measures among others [8]. Compelling evidence from the few studies that have investigated the effect of malaria-helminths co-infections suggests that an interaction between the two diseases might influence the clinical outcome of the involved diseases. However, inconsistencies about the clinical outcome of these interactions on malaria are common, both protective and detrimental effects have been reported [9, 10]. It has been reported previously that helminths increase vulnerability to malaria [11, 12], increase malaria parasitemia [13, 14], with a subsequent increase in malaria disease severity [15, 16].

However, in other related studies no apparent effect of helminths co-infections on malaria risk or disease severity were observed [17–19]. On the contrary, in other studies concurrent helminths infections have been associated with low malaria incidence [20], decreased parasitemia [20, 21], protection from cerebral malaria [22], nephron protective effects [20–22], and a reduction in malaria disease severity [11, 20–24]. Although the underlying mechanisms responsible for these varying responses are not well characterized, compelling evidence suggests that host inflammatory cytokines might be key players.

Malaria infections are generally characterized by a T helper 1 (Th1) response predominated by a number of pro-inflammatory cytokines with a gradual shift to Th2 response as the disease progresses [25, 26]. Pro-inflammatory cytokines like IFN-γ and TNF-α have been demonstrated to play critical roles early in the infection [27, 28]. Conversely, if pro-inflammatory cytokines are not regulated by counter inflammatory cytokines like IL-10 and TGF-β, the resulting pathology is exacerbated [29]. On the other hand, helminths infections are characterized by a strong Th2 immune response [30, 31] dominated by an up-regulation of counter inflammatory cytokines like IL-10 and TGF-β [32, 33]. Therefore, it is likely that during poly-parasitism if the counter balance between the Th1 and Th2 immune responses is not achieved, the clinical course of involved diseases might be altered. It’s proposed that host cytokines might be partly involved in driving the clinical outcome of malaria-helminths co-infections. However, literature on how these interactions influence the host cytokine profiles is still scarce with the few available studies reporting contradictory findings [29]. With such inconsistences, the questions as to whether co-infections impact on malaria incidence, clinical outcome and disease severity have not been conclusively addressed. Furthermore, although cytokine responses have been extensively described in *P. falciparum* and soil borne helminth infection separately [34], few studies have looked at systemic cytokine level concentration in co-infection of malaria and soil borne helminth parasites. We therefore, determined Cytokine profiles (IFN-γ, TNF-α, IL-6, TGF-β and IL-10) associated with *Plasmodium falciparum* malaria and soil borne helminth co-infections among patients attending Kampala International University Teaching Hospital in Uganda.

## Methods

### Study design

A case–control study was conducted at Kampala International University Teaching Hospital Out Patients Department (OPD) in Bushenyi district. Cases were defined as having a positive blood smear for *P. falciparum* malaria, those with helminths or those co-infected with both diseases. Negative controls were those with a negative blood smear for *P. falciparum* malaria and those with no stool parasitic infections. Patients presenting with signs and symptoms of malaria or those suspected of having helminths were recruited for the study. From each patient and control, 5 ml of venous blood was collected into EDTA vacutainers. Malaria diagnosis and parasite quantification was done using Giemsa stained thin and thick blood smears [35]. Stool examination was done following the Kato Katz technique [36] and worm burden categorized following WHO guidelines [37]. For statistical analyses, cases were categorized as those with malaria or helminths alone and those co-infected with both malaria and helminths, these were compared with health controls.

Upon recruitment into the study, a detailed clinical history was sought from each patient. Physical examination of patients was performed by a medical office. Data recorded on the clinical form included the patient’s demographic characteristics, clinical presentation, and perceived onset of illness. Children with other stool parasitic infections, those on anti-helminthic and malarial treatment were excluded from the study.

### Collection of blood samples

For immunological assays, 5 ml blood were collected from each patient in EDTA vacutainers and centrifuged for 10 min at 3000*g* using an Eppendorf centrifuge. Plasma was subsequently aliquoted and stored at − 20 °C in the hospital laboratory refrigerator until further analysis. Samples were later transported in an ice cold box to immunology and molecular biology laboratory at the College of Veterinary Medicine, Animal Resources and Biosecurity (COVAB), Makerere University for cytokine assay analysis.

### Blood smear for malaria diagnosis

Upon recruitment, *P. falciparum* diagnosis was done using thin and thick blood smears stained with 10% Giemsa. Parasite evaluation was done by microscopic examination of 200 fields at 100× magnification and parasite density expressed as number of parasites/µl [35].

### Cytokine assays

Panels of both pro-inflammatory and counter inflammatory cytokines (IFN-, TNF-, TGF-β, IL-6, IL-10,) were assayed from plasma samples using solid phase sandwich enzyme-linked immunosorbent assay (Bio-Rad, UK) assay following manufacturer’s instructions as previously described [38]. All assays were done in triplicate. Values of cytokine concentrations were extrapolated from standard curves obtained from recombinant cytokine standards using Graph Pad version 6 statistical software.

### Stool collection and evaluation

Stool samples were collected in dry leaky proof plastic bottles and approximately 3 g were used. Stool examination was done following the Kato Katz technique [36] and worm burden categorized following WHO guidelines [37].

### Statistical analyses

All data were anonymized prior to analysis using Graph Pad Prism version 6.0. All numerical variables was summarized using mean and standard error of the mean (SEM). Before analysis of cytokine data, deviation from normality was tested using D’Agostino-Pearson normality test. Because none of the cytokines presented a normal distribution, data were presented as medians. Comparisons between groups were done using Kruskal–Wallis non parametric test followed by Dunn’s post-test at a significant level (P < 0.05).

## Results

### Patient’s characteristics

In this study a total of 240 patients were recruited. The mean age of patients was 7.6 ± 3.0 years with a sex ratio of 1.1 (male: female). Patients with *Plasmodium falciparum* malaria were 55 (22.9%) and those with soil-borne helminths were 63 (26.3%). The controls were 89 (37.1%), while those co-infected with *Plasmodium falciparum* malaria and soil-borne helminths were 33 (13.8%) as shown in Table 1. The intensity of *Plasmodium falciparum* malaria by sex was 28 (11.7%) and 27 (11.3%) in male and female respectively. Severe *Plasmodium falciparum* was detected in only 4 (4.5%) patients as compared to those with moderate 8 (9.1%) and low parasite intensity 76 (86.4%). *Plasmodium falciparum* intensity according to sex was 42 (47.7%), 3 (3.4%) and 4 (4.5%) for low, moderate and severe respectively in males and 34 (38.6%), 5 (5.7%) and 0 (0.0%) for low, moderate and severe respectively in females.

**Table 1:** Patient’s baseline characteristics

	Male	Female	Total	P value
Soil-borne helminths	30 (12.5%)	33 (13.8%)	63 (26.3%)	0.539
*Ascaris lumbricoides*	38 (39.6%)	34 (35.4%)	72 (75.0%)	
Hookworms (*Ancylostoma duodenale* and *Necator americanus*)	13 (13.5%)	7 (7.3%)	20 (20.8%)	
*Trichuris trichiura*	0 (0%)	0 (0%)	0 (0%)	
Mixed infections (*Ascaris lumbricoides* + Hookworms)	0 (0%)	4 (4.2%)	4 (4.2%)	
*Plasmodium falciparum* parasitaemia	28 (11.7%)	27 (11.3%)	55 (22.9%)	0.362
Low	42 (47.7%)	34 (38.6%)	76 (86.4%)	
Moderate	3 (3.4%)	5 (5.7%)	8 (9.1%)	
Severe	4 (4.5%)	0 (0.0%)	4 (4.5%)	
Co-infections	21 (8.8%)	12 (5.0%)	33 (13.8%)	0.479
*Ascaris lumbricoides* + *Plasmodium falciparum*	15 (42.3%)	11 (36.5%)	26 (78.8%)	
Hookworms + *Plasmodium falciparum*	6 (14.3%)	1 (6.9%)	7 (21.2%)	

Low (1–10 parasites per 100 thick film fields), Moderate (11–100 parasites per 100 thick film fields), Severe (1–10 parasites per each thick film field)

### Prevalent soil-borne helminths

The overall prevalence of soil-borne helminths was 63 (26.3%). Among those harbouring helminths, 33 (13.8%) were co-infected with *Plasmodium falciparum.* The most prevalent soil-borne helminths observed was *Ascaris lumbricoides,* 72 (75.0%) followed by hookworms, 20 (20.8%). Mixed infection were only of *Ascaris lumbricoides* and hookworms with a prevalence 4 (4.2%). No *Trichuris trichiura* infections were observed in the study population. Among the helminths co-infections, *Ascaris lumbricoides* had the highest prevalence 26 (78.8%) as compared to hookworms 7 (21.2%). No significant differences were observed in soil-borne helminths across sex (P > 0.05, Table 1).

### Cytokine expression in *Plasmodium falciparum* malaria and soil-borne helminths

**Fig. 1:**
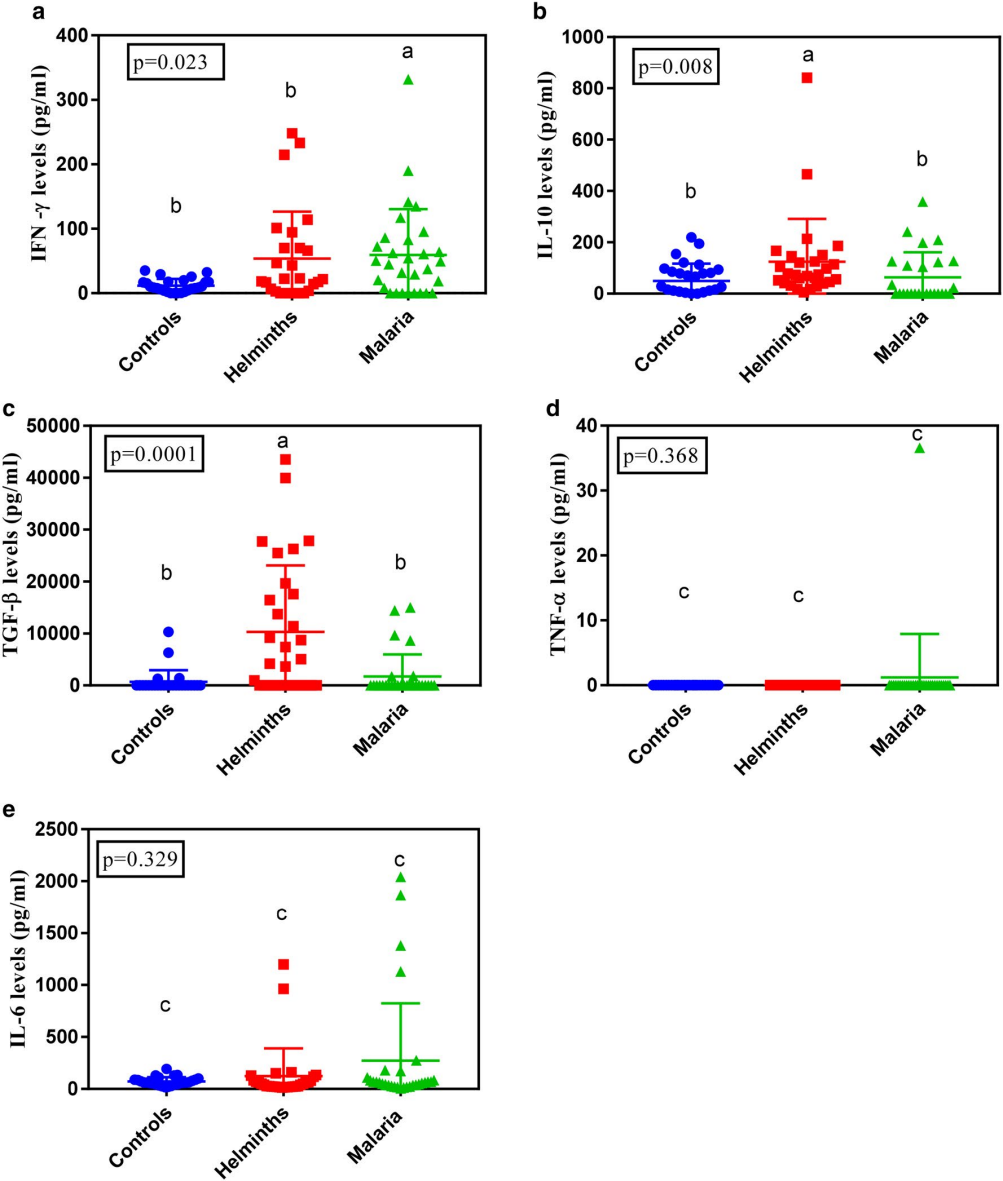
Plasma cytokine profiles in *Plasmodium falciparum* malaria cases, soil borne helminths and normal controls for IFN-γ (**a**), IL-10 (**b**), TGF-β (**c**), TNF-α (**d**) and IL-6 (**e**). Graphs show median level with interquartile range. Letters above the bars indicate significant difference between the groups (Dunn’s post-test, P ≤ 0.05)

In order to identify cytokines that are associated with either *Plasmodium falciparum* malaria or soil borne helminth infections, cytokines from each group of patients were compared with health controls. The results showed that cytokine levels significantly differed across groups for IFN-γ (P = 0.023, Fig. 1a), IL-10 (P = 0.008, Fig. 1b) and TGF-β (P = 0.0001, Fig. 1c). No Significant differences were observed for TNF-α (P = 0.368, Fig. 1d) and IL-6 (P = 0.329, Fig. 1e).

When median plasma IFN-γ, IL-10 and TGF-β levels were compared across groups, the results showed that *Plasmodium falciparum* malaria infected individual expressed significantly higher (P < 0.05) levels of IFN-γ (47.7 pg/ml) as compared to health controls (8.8 pg/ml) and soil borne helminths infected individuals (22.8 pg/ml, Fig. 1a). When IL-10 levels were similarly compared soil borne helminths infected individuals expressed higher levels of IL-10 (73.86 pg/ml) as compared to *Plasmodium falciparum* malaria (33.64 pg/ml) and healthy individuals (26.09 pg/ml, Fig. 1b). When median plasma cytokine levels of TGF-β were compared across groups, the results showed that soil borne helminths infected individuals showed higher levels (P < 0.05) of TGF-β (2338 pg/ml) as compared to *Plasmodium falciparum* malaria infected individuals (772 pg/ml) and healthy controls (424.6 pg/ml, Fig. 1c). No significant differences across groups were noted for TNF-α and IL-6 cytokines levels (Fig. 1d, e respectively).

### Cytokine expression in *Plasmodium falciparum* malaria and soil-borne helminths co-infection

**Fig. 2:**
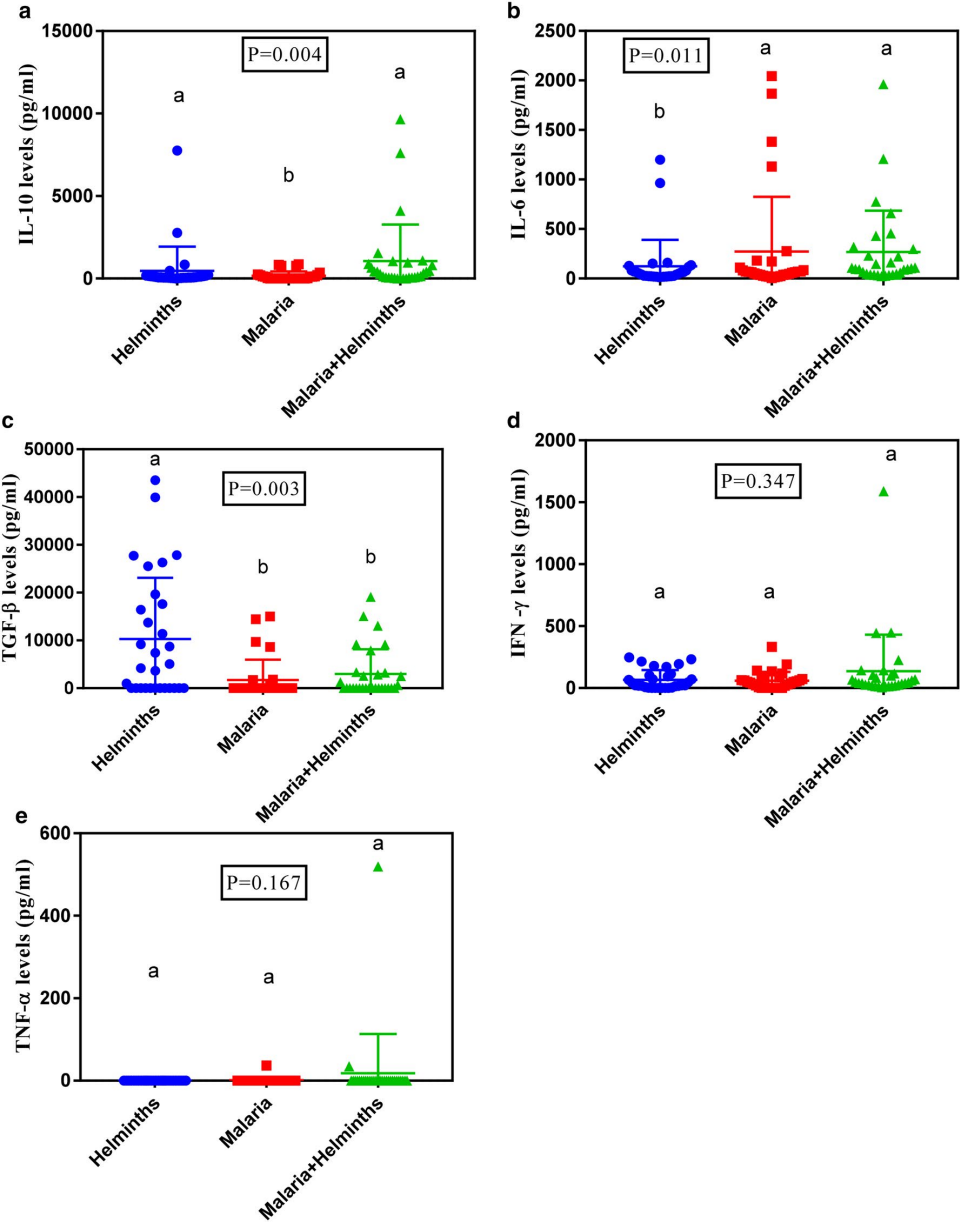
Plasma cytokine profiles in *Plasmodium falciparum* malaria cases, soil borne helminths and co-infections for IL-10 (**a)**, IL-6 (**b**), TGF-β (**c**), IFN-γ (**d**) and TNF-α (**e**). Graphs show median level with interquartile range. Letters above the bars indicate significant difference between the groups (Dunn’s post-test, P ≤ 0.05)

In order to find the effect of harbouring both soil-borne helminths and *Plasmodium falciparum* malaria concurrently, cytokine levels for co-infected individuals were compared with those with either *Plasmodium falciparum* malaria or soil-borne helminths alone. Results showed that cytokine levels significantly differed across groups for IL-10 (P = 0.004, Fig. 2a), IL-6 (P = 0.011, Fig. 2b) and TGF-β (P = 0.003, Fig. 2c). No Significant differences were observed for IFN-γ (P = 0.347, Fig. 2d) and TNF-α (P = 0.167, Fig. 2e).

When median plasma cytokine levels were compared across groups, the results showed that co-infected individuals expressed significantly (P < 0.05) higher levels of IL-10 (304 pg/ml) as compared to *Plasmodium falciparum* malaria infected individuals (11.57 pg/ml) and soil borne helminths infected individuals (77.01 pg/ml, Fig. 2a). When median plasma cytokine levels of IL-6 were compared across groups, the results showed that co-infected individuals showed higher (P < 0.05) levels of IL-6 (100.9 pg/ml) as compared to *Plasmodium falciparum* malaria infected individuals (63.7 pg/ml) and soil borne helminths infected individuals (45.37 pg/ml, Fig. 2b). Similarly, when TGF-β levels were similarly compared soil borne helminths infected individuals expressed higher (P < 0.05) levels of TGF-β (2338 pg/ml) as compared to *Plasmodium falciparum* malaria (772.7 pg/ml) and co-infected individuals (939.2 pg/ml, Fig. 2c). No significant differences across groups were noted for IFN-γ and TNF-α cytokines levels (Fig. 2d, e respectively).

## Discussion

The over prevalence of soil borne helminth was (26.3%). This is in range with what has been reported previously in Uganda [39], Tanzania [40] and Ethiopia [4]. Prevalence studies of soil borne helminths since 2009 have indicated that of *Ascaris lumbricoides*, and the hookworm species are common infections in Bushenyi district among the soil borne helminths [39]. This is in agreement with our finding were *Ascaris lumbricoides* had the highest prevalence (75.0%) followed by hook worms (20.8%). In this study no *Trichuris trichiura infections* were detected, similarly to what was observed by Agwu et al. [39] in Bushenyi district. In this study, the *Plasmodium falciparum* malaria prevalence was (22.9%) lower than that reported in previous studies in Uganda 27% [2]. This could probably be because of the difference in the sample size. However, our findings compare well with the earlier report from southwest Uganda [19] that reported a prevalence of 23%. Results from this study are in range with a previous study by Shapiro et al. [19] who reported a prevalence of 15% among malaria helminth co-infected individuals in Uganda, 14% was reported in Zambia [35, 41] and 13% was reported in Kenya (Rutagwera et al. [6]). However this is not in range with a similar study done in Tanzania where they reported a prevalence among malaria helminth co-infected to be 26% [6]. Among the helminths co-infections, *Ascaris lumbricoides* had the highest prevalence (78.8%) and results from this study are in range with other studies done elsewhere in Madagascar which reported that among the helminths co-infections, *Ascaris lumbricoides* had the highest prevalence of 77% [14]. In our study*, Plasmodium falciparum* malaria infected individual expressed significantly higher levels of IFN-γ (47.7 pg/ml) as compared to health controls (8.8 pg/ml) and soil borne helminths infected individuals (22.8 pg/ml). This is in agreement with a study done in south East Asia and elsewhere which reported that *Plasmodium falciparum* malaria expressed higher IFN-γ levels (Doolan et al. [27]; Luty et al. [42]). These higher levels of IFN-γ have been associated with protective role of the cytokine against *P. falciparum* malaria [27, 35]. This effect has been attributed to the monocyte-macrophage activating capacity of IFN-γ, with rapid killing of the malarial blood-stage parasites by reactive oxygen and nitrogen intermediates [43, 44]. Also animal models of malaria have been associated with increased production of IFN-γ which has been associated with a favourable outcome of the disease [26]. Soil borne helminths infected individuals expressed higher levels of IL-10 (73.86 pg/ml) as compared to *Plasmodium falciparum* malaria (33.64 pg/ml) and healthy individuals (26.09 pg/ml). This is probably because IL-10 is a key anti-inflammatory cytokine protective against helminth infections that cause inflammation [45]. Also soil borne helminths infected individuals expressed the highest levels of TGF-β (2338 pg/ml) as compared to *Plasmodium falciparum* malaria infected individuals (772 pg/ml) and healthy controls (424.6 pg/ml). This is probably because TGF-β has been attributed to wound healing of the intestinal mucosa during tissue repair caused by helminths in the gut [32, 46]. TNF-α and IL-6 levels showed no significant difference across groups and this is in agreement with previous studies done by Franca and colleagues that reported that TNF-α and IL-6 cytokines are not associated with *Plasmodium falciparum* malaria and soil borne helminths infected individuals [47]. This is probably because the TNF-α is a key cytokine in cerebral malaria [48] and probably the patients we examined that time none had cerebral malaria. Also the higher levels of IL-10 and TGF-β expressed in soil borne helminths infected individuals could have down regulated the production of TNF-α levels [30, 49]. Lack of significant difference across groups in IL-6 could probably be because the IL-6 is not involved in modulation of the disease both in *Plasmodium falciparum* malaria infected individuals and soil borne helminths infected individuals. Co-infected individuals expressed significantly higher levels of IL-10 (304 pg/ml) as compared to *Plasmodium falciparum* malaria infected individuals (11.57 pg/ml) and soil borne helminths infected individuals (77.01 pg/ml). Results from this study are in agreement with a previous study by Hartgers who reported high expression of IL-10 in co-infected individuals [50]. Up regulation of IL-10 in co-infections is probably indicative that co-infected individuals don’t lose the protective role of the IL-10 against soil borne helminths during infection. This is in agreement with the role of gut helminth in modulation of TH2 cytokine like responses that lead to worm expulsion [51, 52]. Co-infected individuals expressed higher levels of IL-6 (100.9 pg/ml) as compared to *Plasmodium falciparum* malaria infected individuals (63.7 pg/ml) and soil borne helminths infected individuals (45.37 pg/ml). This is because IL-6 has been shown to possess both pro and anti-inflammatory features [53, 54]. Soil borne helminths infected individuals expressed higher levels of TGF-β (2338 pg/ml) as compared to *Plasmodium falciparum* malaria (772.7 pg/ml) and co-infected individuals (939.2 pg/ml).This down regulation of TGF-β in co-infections is indicative that co-infected individuals lose the protective role of TGF-β against soil borne helminths during infection. IFN-γ and TNF-α levels showed no significance across groups. This is probably because the higher levels of IL-10 in co-infections and soil borne helminths down regulates IFN-γ by supressing T cells from producing IFN-γ hence the decrease [24, 44]. Also TGF-β may down regulates production of TNF-α in soil borne helminths [33]. And lastly TNF-α is probably not involved in modulation of malaria, soil borne helminth and co-infections of both diseases.

## Conclusion

In conclusion, our study demonstrates an up-regulation of IFN-γ during *Plasmodium falciparum* malaria and an up-regulation of IL-10 and TGF-β during soil borne helminth infections. We further show an up-regulation of IL-10 and IL-6 during co-infections of *Plasmodium falciparum* and soil borne helminth infections and a down-regulation of TGF-β during co-infections of *Plasmodium falciparum* and soil borne helminth infections. However, how these cytokines may influence the clinical outcome of the diseases involved calls for further investigations.

## Abbreviations

µl: micro litre
H_2_SO_4_: hydrogen Sulphuric Acid
HCL: hydrochloric acid
IFN: interferon
Ig: immuno globulin
IL: interleukin
KIU-TH: Kampala International University Teaching Hospital
NAOH: sodium hydroxide
nm: nano meter
OD: optical density
P: *Plasmodium*
PBS: phosphate buffered saline
RT: room temperature
SBH: soil-borne helminths
TGF-β: transforming growth factor Beta
Th: T helper
TNF-α: tumor necrosis factor alpha
